# Supplementary material for: The interactome of intact mitochondria by cross-linking mass spectrometry provides evidence for coexisting respiratory supercomplexes
Source: Mol Cell Proteomics. 2017 Dec 8;17(2):216–32. doi: 10.1074/mcp.RA117.000470 (PMC5795388; doi:10.1074/mcp.RA117.000470)
Supplement: Supplemental Data [file supp_RA117.000470_133922_0_supp_23978_fzffwf.zip › spectra_annotation/mito_spectra_annotation/495-1-5-1-8-1.pdf]

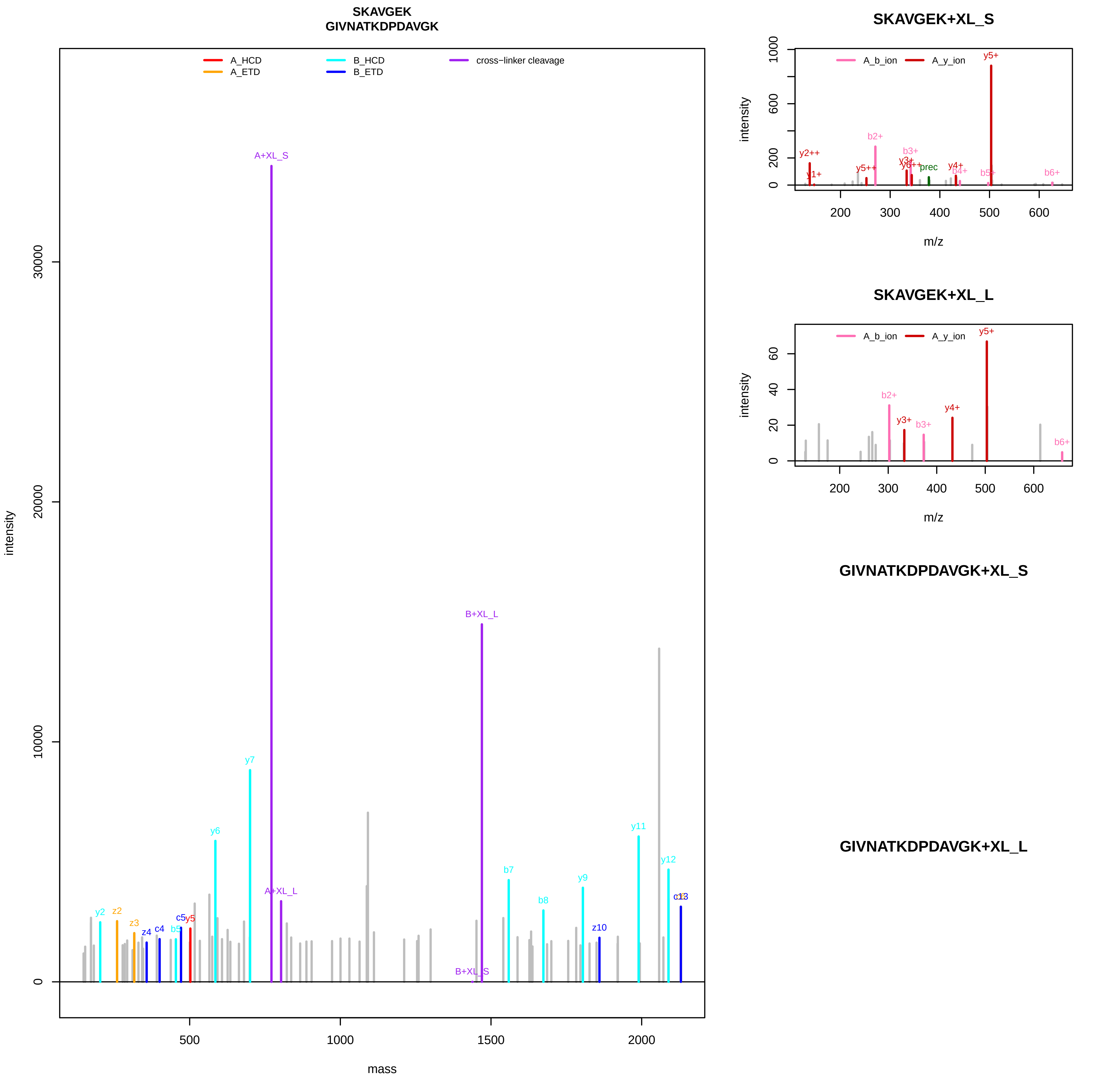
SKAVGEK
GIVNATKDPDAVGK
A_HCD
A_ETD
B_HCD
B_ETD
cross−linker cleavage
A+XL_S
B+XL_L
A+XL_L
B+XL_S
y2
z2
z3
z4
c4
c5
b5
y5
y6
y7
b7
b8
y9
z10
y11
y12
c13
intensity
mass
0
10000
20000
30000
500
1000
1500
2000
SKAVGEK+XL_S
A_b_ion
A_y_ion
y2++
y1+
y5++
b2+
b3+
y3+
y6++
prec
y4+
b4+
b5+
y5+
b6+
0
200
600
1000
200
300
400
500
600
m/z
SKAVGEK+XL_L
A_b_ion
A_y_ion
b2+
y3+
b3+
y4+
y5+
b6+
0
20
40
60
GIVNATKDPDAVGK+XL_S
GIVNATKDPDAVGK+XL_L
